# Short-Chain Flavor Ester Synthesis in Organic Media by an *E. coli* Whole-Cell Biocatalyst Expressing a Newly Characterized Heterologous Lipase

**DOI:** 10.1371/journal.pone.0091872

**Published:** 2014-03-26

**Authors:** Guillaume Brault, François Shareck, Yves Hurtubise, François Lépine, Nicolas Doucet

**Affiliations:** 1 INRS-Institut Armand-Frappier, Université du Québec, Laval, Québec, Canada; 2 Innu-Science Canada, Inc., Local 119, Trois-Rivières, Québec, Canada; 3 PROTEO, the Québec Network for Research on Protein Function, Structure, and Engineering, Québec, Canada; 4 GRASP, Groupe de Recherche Axé sur la Structure des Protéines, Québec, Canada; Queen’s University Belfast, United Kingdom

## Abstract

Short-chain aliphatic esters are small volatile molecules that produce fruity and pleasant aromas and flavors. Most of these esters are artificially produced or extracted from natural sources at high cost. It is, however, possible to ‘naturally’ produce these molecules using biocatalysts such as lipases and esterases. A gene coding for a newly uncovered lipase was isolated from a previous metagenomic study and cloned into *E. coli* BL21 (DE3) for overexpression using the pET16b plasmid. Using this recombinant strain as a whole-cell biocatalyst, short chain esters were efficiently synthesized by transesterification and esterification reactions in organic media. The recombinant lipase (LipIAF5-2) showed good affinity toward glyceryl trioctanoate and the highest conversion yields were obtained for the transesterification of glyceryl triacetate with methanol. Using a simple cetyl-trimethylammonium bromide pretreatment increased the synthetic activity by a six-fold factor and the whole-cell biocatalyst showed the highest activity at 40°C with a relatively high water content of 10% (w/w). The whole-cell biocatalyst showed excellent tolerance to alcohol and short-chain fatty acid denaturation. Substrate affinity was equally effective with all primary alcohols tested as acyl acceptors, with a slight preference for methanol. The best transesterification conversion of 50 mmol glyceryl triacetate into isoamyl acetate (banana fragrance) provided near 100% yield after 24 hours using 10% biocatalyst loading (w/w) in a fluidized bed reactor, allowing recycling of the biocatalyst up to five times. These results show promising potential for an industrial approach aimed at the biosynthesis of short-chain esters, namely for natural flavor and fragrance production in micro-aqueous media.

## Introduction

Flavors and fragrances are broadly exploited molecules in the food, cosmetic, detergent, chemical and pharmaceutical industries, with an estimated global market exceeding $US 22 billion per year [1]. Most flavors are either artificially produced using chemical synthesis or extracted from natural sources [2]. Chemical synthesis often relies on harsh procedures requiring toxic solvents and high-energy transformations, resulting in environmentally damaging processes with a very high carbon footprint. These approaches also suffer from the lack of substrate selectivity, creating racemic mixtures with undesired side products that reduce synthesis efficiency and increase production costs [3]. Extraction of flavors from natural raw materials is also challenging as the targeted molecules are often in very low concentration, thus drastically increasing production costs. Nonetheless, there is a growing interest for the bio-production of these compounds from microorganisms, either using biosynthesis or bioconversion methods. The US and European regulations stipulate that flavors obtained from microbial or enzymatic processes can be considered as natural compounds as long as the initial raw material employed is from a natural source. The possibility of adding a ‘natural’ label to a biotechnologically produced molecule is therefore very attractive, as there is a growing demand for greener products in the general public. The most cited example illustrating this fact is the 100-fold increase in gross retail price of naturally labeled vanillin relative to its synthetic, yet chemically identical counterpart [4]–[6].

Bio-production of flavors can be performed under mild conditions, leading to the synthesis of highly regio- and stereo-specific, optically pure end-products [3]. The two main approaches for the bio-production of flavors are the *de novo* biosynthesis from live organisms and the bioconversion of precursors *via* enzymatic routes. Commercial successes using microorganisms have allowed the annual production of several tons of rare molecules, such as γ-decalactone, a powerful and tenacious peach-like aroma [4], [6]. Using a recombinant auxotrophic *Yarrowia lipolytica* strain, ricinoleic acid could be efficiently converted to γ-decalactone with yields up to 9.5 g/L in 75 h [7]. However, performing bioconversion with live cells presents some limitations, such as slow growth and substrate/product toxicity. As a result, yields are often very low (rarely above 1 g/L), an issue that can only be circumvented *via* elaborate *in situ* extraction techniques such as gas stripping [3], [8]. In some cases, however, *de novo* synthesis of relatively simple molecules is not a prerequisite, and taking advantage of pure or crude enzyme preparations to synthesize flavors can be much more efficient [9]. Enzymes are often more expensive than chemical catalysts, but techniques such as immobilization allow recycling of the biocatalyst and help reduce production costs [10]. More recently, up to 400 g/L of the fragrance compound 2(Z)-methyl-5-isopropyl-2,5-hexadianal (isonovalal) were produced in only 2.5 h using permeated whole cells of *Pseudomonas rhodesia* CIP 107491 [11]. These results demonstrate that the use of whole-cell biocatalysts (WCB) is an industrially simple and cost-effective approach to enzyme immobilization [12], [13].

Short- and medium-chain fatty acid esters are commonly used as important flavoring and fragrance molecules because of their typical fruity smell and high volatility [6], [14]. Enzyme-catalyzed esterification is an effective alternative to the chemical synthesis of short-chain esters. Esterases and lipases (triacylglycerol hydrolases; EC 3.1.1.3) are important industrial enzymes with great potential for the production of flavors and fragrances [15], [16]. These biocatalysts generally do not require cofactors and are stable in organic solvents, thus facilitating the synthesis of hydrophobic or water-labile compounds [17]. In non-aqueous conditions, lipases typically catalyze the esterification, interesterification, and transesterification of alcohols and fatty acids, the three main chemical reactions producing flavoring esters [16]. Lipases can also resolve racemic mixtures in a regio- and/or enantioselective fashion, such as the complete resolution of *D*,*L*-methylmenthol esters into pure *L*-menthol [18].

Although lipolytic enzymes are found in all living organisms, most commercial enzymes originate from microbial sources [16], [19]. As a result, bacterial genome mining of versatile microorganisms offers an attractive opportunity to uncover new lipolytic biocatalysts displaying interesting biochemical properties for industrial applications. To that end, one of the most powerful approaches relies on functional metagenomics of enriched biomass samples [20], [21]. Several genes coding for genuine lipases have been successfully achieved using this approach [21]–[23]. Nevertheless, expression and production of promising biocatalysts is often poor and hazardous, yielding few industrial applications. As a result, several attempts have been made to improve the production of enzyme biocatalysts, including intracellular accumulation of lipases in the form of WCB [24], [25]. Such production allows an enzyme to be used without complex procedures for isolation, purification, or immobilization, with the added benefit that whole cells act as a protective environment for biosynthesis [26]. Recently, Wei and collaborators successfully produced long-chain methyl esters using a heterologously expressed *Proteus* sp. lipase in permeated *E. coli* whole cells [27]. Such a simple yet effective WCB was also employed for the racemic resolution of *D*,*L*-menthyl esters with a *Bacillus subtilis* ECU0554 lipase, demonstrating that the system can be applied to various lipolytic enzymes and bacterial systems [28].

We have recently reported the characterization of novel lipases from metagenomic studies [22], [23]. One such lipase, LipIAF5-2, was heterologously expressed in the actinomycete *Streptomyces lividans* and showed promising short- and long-chain specificity while being solvent-tolerant and thermostable [22]. However, standard expression yields were too low to sustain any relevant industrial application. In the present work, we report the cloning and expression of this lipase as an *E. coli* whole-cell biocatalyst, showing that the resulting construct is a relevant WCB for the organic synthesis of important fatty acid esters in micro-aqueous media.

## Materials and Methods

### Materials, Bacterial Strains, and Plasmids

All triglyceride substrates were purchased from Sigma-Aldrich Canada (Oakville, ON) using the highest purity available. Analytical standards for methyl acetate, isoamyl acetate, methyl butyrate, methyl hexanoate, methyl octanoate, ethyl butyrate, propyl butyrate, butyl butyrate and amyl butyrate were also purchased from Sigma-Aldrich. Analytical grade methyl nonanoate was purchased from Chromatographic Specialties (Brockville, ON). All other chemicals were obtained commercially and were of analytical grade.

The strain *Escherichia coli* DH11S was employed for all subcloning procedures. The strain *E. coli* BL21 (DE3) was employed for the heterologous expression of the lipase gene. Expression vector pET16b (EMD Millipore) was used for the production of the N-terminal histidine-tagged recombinant protein. Plasmid pIAFD95A was used as template for the amplification of the *lipIAF5-2* gene (Genbank EU660533) [22].

### DNA Manipulations

The recombinant gene coding for lipase LipIAF5-2 was amplified without signal peptide (from Ala^28^ [22]) using primers F5_2_NdeI 5′-AAACATATGGCCAACCCGCCGGGGGGCGATCC-3′ and F5_2_XhoI 5′-TTTCTCGAGTCAGTACGGGCAGTTACCTCGGTATTCGGAG-3′. Restriction sites were added to both ends (5′-*Nde*I and 3′-*Xho*I, both underlined) to allow subcloning in expression vector pET16b. PCR-amplified fragments were cleaned using the Qiagen PCR cleaning kit, cloned into the *Nde*I/*Xho*I-digested pET16b, and transformed into *E. coli* DH11S, yielding the pET16b-F52 construct used for protein expression. The integrity of both plasmid strands was confirmed by DNA sequencing using forward and reverse T7 universal primers (Génome Québec).

### Heterologous Expression and Purification of LipIAF5-2

The heterologous expression and purification of LipIAF5-2 from *E. coli* BL21 (DE3) was performed as recently described using a Ni-NTA (Qiagen) affinity column [29]. All cultures were inoculated from fresh Petri cultures following bacterial transformation with the sequenced constructs. Analysis of the purified proteins was performed by standard SDS-PAGE procedures. Western Blot and Zymogram analyses were employed to confirm the activity of the recombinant LipIAF5-2 at each purification step. The presence of the recombinant protein was revealed using femtoCHROMO-HRP (G-Biosciences) following the manufacturer’s instructions. The identity of the protein was confirmed by mass spectrometry, as previously described [29], [30].

### Preparation of the *E. coli* Whole-cell Biocatalyst

*E. coli* BL21 (DE3) harboring construct pET16b-F52 was grown in Erlenmeyer flasks as previously described and cells were treated as published, with minor variations [27]. Cell pellets were washed twice with cold physiological saline buffer (PBS, pH 7.0) prior to suspension in PBS supplemented with 0.3% cetyl-trimethylammoniumbromide (CTAB) for 60 min at room temperature with gentle agitation. Treated cells were centrifuged and washed once with PBS prior to lyophilization. Cells were then used as a dry powder biocatalyst.

### Enzyme Assays

#### Standard hydrolysis assays and enzyme kinetics

Standard assays were performed by measuring the rate of triglyceride hydrolysis using a previously described pH-stat method [29]. The fatty acids released from emulsions of the triacylglycerides (TAG) glyceryl triacetin (C2∶0), glyceryl tributyrin (C4∶0), glyceryl trioctanoate (C8∶0), and glyceryl triolein (C18∶1) were automatically titrated using a 0.05 M NaOH solution to maintain a constant pH of 9.0 at 40°C in a Metrohm 800 Dosino apparatus (Mississauga, ON). One unit (U) was defined as the amount of enzyme releasing 1 μmol of fatty acid per minute in the conditions described. The effect of temperature on enzyme activity was determined by assaying the hydrolytic activity towards glyceryl trioctanoate using the same procedure. Enzyme kinetics were performed with dilutions of the purified lipase and various triglyceride substrates at concentrations varying from 0.1 to 100 mM at the optimal temperature of 40°C. The catalytic rate constant *k*_cat_ (s^−1^) was calculated from the initial steady-state velocity according to the equation *k*_cat_ = V_max_/[E].

#### Esterification and transesterification assays

Standard transesterification assays were conducted using 1 mmol of the glyceryl tributyrate substrate, 4 mmol methanol and 5% water (w/w) in a final volume of 1 mL completed with *tert*-butanol. The mixture was preheated and added to preheated conical-bottom screw-cap microvials (1.15 mL) containing 10 mg of the dry whole-cell biocatalyst to start the reaction. The reaction was carried out at the optimal temperature of 40°C and 1400 rpm using a thermomixer. At each predetermined time interval, a 25 μl aliquot was withdrawn with a Hamilton gastight syringe and diluted 1∶20 in 2-propanol to quench the reaction. Methyl nonanoate was added as an internal standard and the samples were centrifuged at 15,000 *g* for 5 min prior to transfer into microvials for HPLC analysis. To evaluate the effect of water on ester production, 1 mmol of glyceryl tributyrate was dissolved as previously mentioned and the water content was adjusted from 0% to 75% (w/w). The reaction was carried out at the optimal temperature of 40°C. Comparison between esterification and transesterification reactions was performed with 1 mmol of butyric acid or glyceryl tributyrate under the conditions described above, adding 100 mg of a 3-Å molecular sieve to withdraw water produced during the esterification reaction. The effect of acyl chain length on esterification was tested using 4 mmol of methanol with 1 mmol of acetic, butyric, hexanoic, or octanoic acid in the standard assay conditions. The effect of alcohol as acyl acceptor for transesterification was tested using 1 mmol of glyceryl tributyrate and 4 mmol of either methanol, ethanol, 1-propanol, 1-butanol, or pentyl alcohol. The effect of co-solvent on synthetic activity was carried out using the same standard assay using either acetonitrile or *tert*-butanol as water-soluble polar solvents, ethyl acetate or 2-octanol as hydrophobic polar solvents, and *n*-hexane or *n*-heptane as hydrophobic non-polar solvents.

### HPLC Analysis

Samples (10 μl) were loaded onto a Phenomenex Synergi Hydro-RP C-18 silica-based column (150×4 mm) (Torrance, CA) using an isocratic mobile phase of 70% acetonitrile, 5% methanol, 25% water, and 0.2% formic acid (v/v) at a flow rate of 1 ml/min and 40°C. Esters produced were detected at 210 nm and identified by comparing retention times of pure analytical standards. Quantification was performed by dividing the slope of standard curves by the measured area under the curve for each identified compound.

### Fluidized Bed Reactor for Flavor Ester Production

A fluidized bed reactor (FBR) was built using an empty HPLC preparative column (10 cm^3^ volume, 21.5 mm diameter). A 0.22-μm Anodisc 25 Whatman filter was placed over both frits to avoid cell leaching and filters were covered with a 25-mm rubber O-ring to ensure a leak-proof assembly. One gram of lyophilized WCB (approximately 7 cm^3^ g^−1^) was loaded on the column and cells were settled by pumping through warm *tert*-butanol. The flow was directed to keep cells in suspension and to avoid any build-up at the filters, thus creating a FBR. The apparatus was placed into a heated chamber at 35°C and the reactions were started by pumping a pre-warmed mixture of 50 mmoles of glyceryl tributyrate, 250 mmoles of isoamyl alcohol, 10% water (w/w) and *tert*-butanol to a final volume of 50 mL (1 mL/min flow rate). 50-μL aliquots were taken at different time intervals over a period of 24 hours and analyzed by HPLC for ester content. Between each synthesis cycle, the FBR column was washed with 300 mL of warm *tert*-butanol at 1 mL/min.

## Results and Discussion

### Recombinant Expression and Purification of LipIAF5-2

Short chain esters are pleasant smelling molecules usually produced using harsh chemical routes and/or laborious extraction steps from natural feedstock. Using biocatalysts such as lipases for their chemical synthesis could help overcome these issues, in addition to providing the commercialized products with the highly sought ‘natural’ label [31], [32]. The need for novel enzymes such as lipases and esterases with interesting biochemical properties is in high demand and metagenomic studies have recently proven resourceful for their discovery [20], [33]. We previously expressed the recombinant LipIAF5-2 isolated from a metagenomic study in the versatile Gram-positive *Streptomyces lividans* [22], [23]. While the protein was produced as an active extracellular form in this organism, expression yields were too low for industrial applications. Herein, we focus on an intracellular expression model of the LipIAF5-2 lipase using an *E. coli* whole-cell biocatalyst (WCB). The industrial use of esterases and lipases requires an easy overexpression system that can produce very high yields of active enzyme in a short amount of time. Furthermore, the industrial use of biocatalysts often requires enzyme immobilization to facilitate large-scale production and to reduce costs. Still, the price of immobilized biocatalysts remains a bottleneck for these biocatalysts to be considered a profitable alternative to chemical synthesis [10]. Using whole-cell biocatalysts offers numerous advantages over free enzymes and represents a cost-effective alternative for a number of industrial applications [13]. To attain this goal, we developed an *E. coli* recombinant system that can achieve high yields of LipIAF5-2 from simple LB medium growth at 16°C in only 24 hours. Under theses conditions, the enzyme was overproduced as a cytoplasmic, soluble biocatalyst with yields exceeding 350 mg/L. High yields of intracellular LipIAF5-2 expression provide a strong basis for the development of an industrial WCB, potentially eliminating the need for cell lysis and protein purification steps altogether. Nevertheless, LipIAF5-2 was also purified to near homogeneity from a fast, single-step immobilized metal affinity chromatography (IMAC) with an average yield of 50±2 mg (average of three independent lots), confirming that the enzyme is an attractive candidate for large-scale production (Fig. S1A). Two endogenous lipolytic enzymes were present in the *E. coli* strain with control plasmid, both of which were further efficiently discarded by chromatography (Fig. S1B). The recombinant LipIAF5-2 protein showed an apparent molecular weight of 32 kDa, consistent with the expected theoretical weight of 32.6 kDa (including the His-tag). SDS-PAGE showed no protein contaminant and the identity of the protein was confirmed with anti-His-tag Western Blot analysis. The identified protein was further digested with trypsin for peptide sequencing using mass spectrometry with Mascot analysis. Our results confirmed that only the GenBank entry ACC95208.1 was obtained, with 62% sequence coverage and an MOWSE score of 785, providing convincing evidence of the purified protein identity. Mass spectrometry analysis of the purified protein confirmed a total protein mass of 32,564 Da, corresponding to the theoretical mass of the mature His-tagged protein (starting from Ala^28^) plus 14 Da, which may result from a post-translational arginine or lysine mono-methylation.

#### Kinetics of LipIAF5-2 toward triglycerides

**Figure 1:**
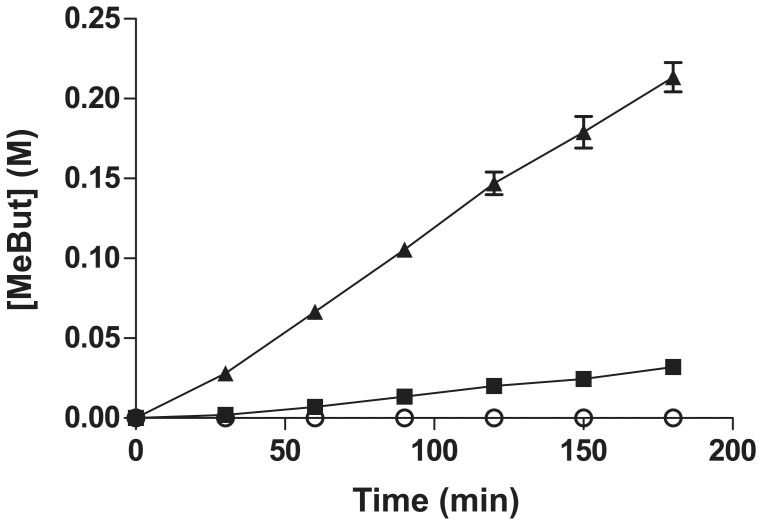
Effect of whole-cell biocatalyst (WCB) pretreatment on ester synthesis activity. Ten milligrams of each biocatalyst was used to convert 1% (w/w) water in *tert-*butanol over a period of 3 hours at 40°C. The control strain expressing the empty pET16b vector (open circles), the LipIAF5-2 strain without CTAB pretreatment (closed squares) and the strain with CTAB permeation (closed triangles) were employed. Results are averaged over three independent experiments.

To evaluate the potential of LipIAF5-2 for short chain ester synthesis, the kinetic parameters of the purified enzyme were assessed for hydrolysis of various short chain TAGs (C2∶0, C4∶0, C8∶0) and one long chain TAG (C18∶1). Kinetic parameters for LipIAF5-2 are reported for substrate concentrations where the enzyme displays Henri-Michaelis-Menten behavior (Table 1). Results for glyceryl triacetate are not listed, as the apparent kinetics of the enzyme could not reach a saturation point in the range of substrate concentrations tested. Higher substrate concentrations could not be achieved without impairing other parameters and were therefore discarded. The calculated *k*_cat_ values decrease as the acyl chain length increases for substrates C4∶0 to C18∶1, but the highest catalytic efficiency (*k*_cat_/*K*_m_) was obtained for glyceryl trioctanoate, a mid-chain long substrate. This result is primarily caused by an 8.6-fold and 2.1-fold greater affinity (*K*_m_) of LipIAF5-2 for C8∶0 relative to C4∶0 and C18∶1 substrates, respectively. These results are similar to the hydrolysis profile of the previously described enzyme characterization with *p*-nitrophenyl esters [22]. Still, the enzyme showed good catalytic efficiency toward the glyceryl trioleate substrate (C18∶1), thus confirming its true lipase nature [19]. The free and purified recombinant enzyme expressed from *E. coli* had a strong tendency to precipitate after purification, probably due to the very high expression yields obtained with this bacterial system. The natural tendency of lipases for self-aggregation due to their surface hydrophobicity could also explain such behavior [34]. Complementation with various additives such as detergents proved unsuccessful at minimizing LipIAF5-2 denaturation. Therefore, we decided to use the recombinant cell as a carrier to circumvent this problem, allowing enzyme activity, stability and immobilization at once. Preliminary tests of the WCB showed that a control strain expressing the empty vector had no synthetic activity, while the recombinant LipIAF5-2 strain was efficient for methyl butyrate (MeBut) synthesis (Fig. 1). It is known that the outer membrane of Gram negative bacteria is very selective toward most hydrophobic molecules [35]. Therefore, we decided to circumvent this limitation using a simple permeabilization step with the cationic surfactant CTAB prior to lyophilisation of the WCB [27]. This simple pre-treatment increased the synthesis activity by a six-fold factor (Fig. 1). Previous WCB reports highlighted similar observations and recommended cell permeation to cope with mass transfer issues across cell membranes [6], [11], [24], [27], [36].

**Table 1:** Kinetic parameters of recombinant LipIAF5-2 toward short chain triglycerides.

Substrate	*K*_m_ ± SD^a^	*k*_cat_	*k*_cat_ */K*_m_
	(mM)	(s^−1^)	(s^−1^ mM^−1^)
Glyceryl tributyrin (C4∶0)	5.46±0.47	2.9×10^4^	5.3×10^3^
Glyceryl trioctanoate (C8∶0)	0.63±0.01	2.3×10^4^	3.7×10^4^
Glyceryl trioleate (C18∶1)	2.47±1.0	1.1×10^4^	4.5×10^3^

a Standard deviation was derived from two different experiments, each performed in triplicate.

### Effect of Temperature on WCB

**Figure 2:**
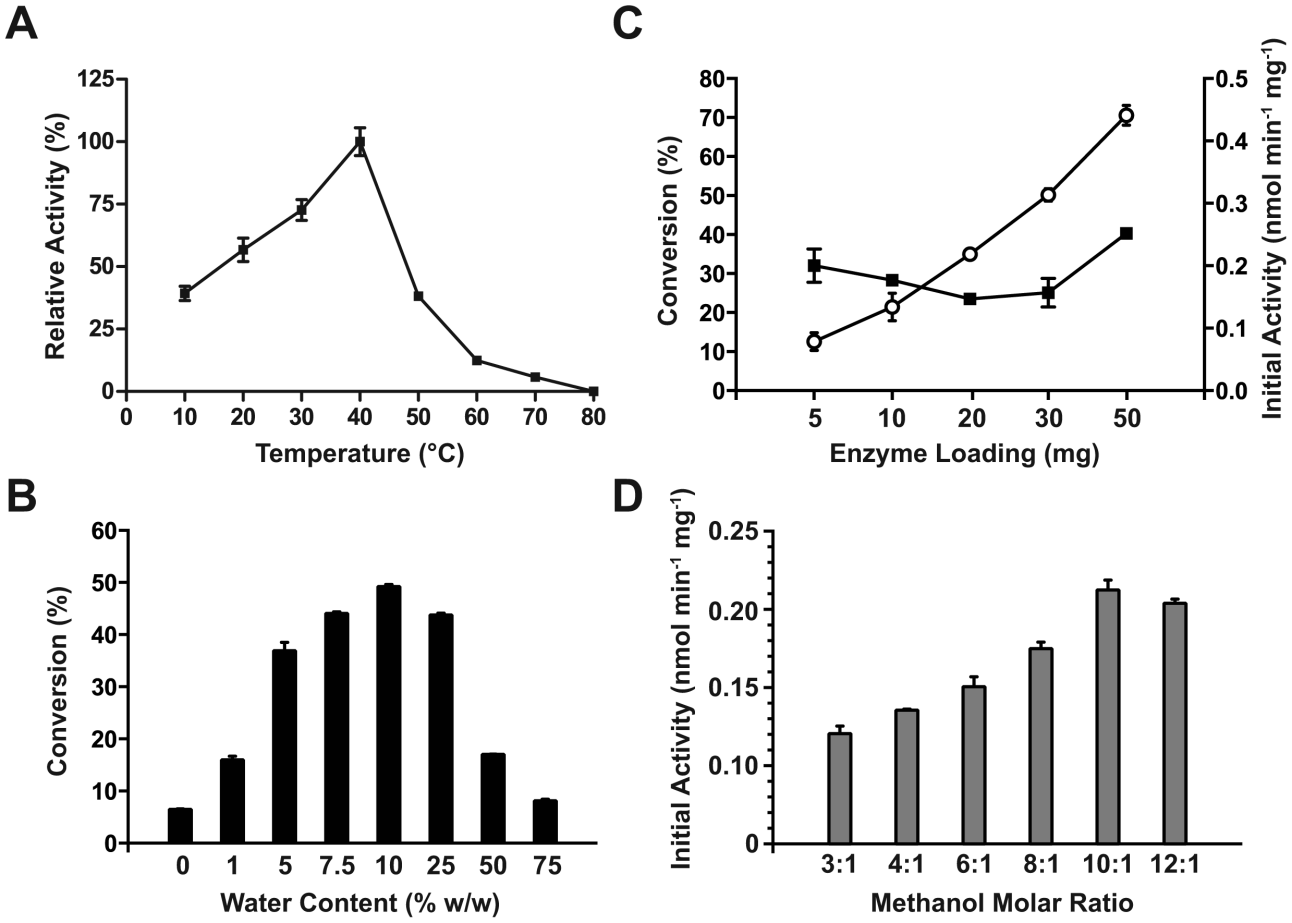
Effect of temperature (A), water content (B), enzyme loading (C), and methanol molar ratio (D) on the LipIAF5-2 WCB activity. A) Effect of temperature on the hydrolytic activity of the LipIAF5-2 WCB. Enzyme activities are expressed relative to maximal activity observed at 40°C (full squares). B) Effect of water content by percentage of substrate weight. Enzyme activities are expressed as final conversion of 1 mmol of glyceryl tributyrate and 4 mmol of methanol after 6 hours of incubation at 40°C. C) Effect of enzyme loading on the conversion of 1 mmol of glyceryl tributyrate using 4 molar equivalents of methanol as acyl acceptor in the aforementionned conditions. Conversion of glyceryl tributyrate (open circles) is expressed as final percentage of theoretical maximal yield (3 mmoles of product) after 3 hours of reaction. Initial specific activity (full squares) is expressed as the slope of liberated product determined under linear conditions over a period of at least 30 minutes. Biocatalyst amounts correspond to 1.65, 3.30, 6.61, 9.92 and 16.53% of substrate weight, respectively. D) Effect of alcohol molar ratios on the LipIAF5-2 WCB initial activity. Initial activity was determined in linear conditions over a 3-hour period using 10 mg of WCB, 5% water (w/w), 1 mmol of glyceryl tributyrate and various molar ratios (alcohol/substrate) of methanol in a final volume of 1.5 mL.

The effect of temperature on LipIAF5-2 WCB was determined by monitoring the hydrolytic activity toward glyceryl trioctanoate at various temperatures from 10 to 80°C (Fig. 2A). Maximum activity was observed at 40°C, with a drastic decrease observed above this temperature. This optimal temperature is different from the 60°C optimal temperature previously reported with the free wild-type enzyme [22]. This discrepancy could be explained by the fact that we are using the truncated and tagged form of LipIAF5-2 (mature enzyme without signal peptide), and by the different substrates tested in both reports (triglyceride *vs*. *p*-nitrophenyl esters). Indeed, it is documented that various substrates can shift the observed optimal temperature of enzymes by creating protective effects on their thermal denaturation [37]. These results nevertheless suggest that the LipIAF5-2 WCB could be useful for ester synthesis at low- to mid-range temperatures.

Immobilized lipases generally show similar or higher optimal temperatures relative to their free counterparts [38]. As a result, the thermal stability of LipIAF5-2 was also assayed with a pre-incubation time of 3 hours at various temperatures in an aqueous buffer. Measurement of residual hydrolysis toward glyceryl trioctanoate showed a WCB thermal denaturation profile identical to that of the free enzyme (data not shown). While we did observe that cell immobilization considerably increased the thermostability of LipIAF5-2 in organic media (not shown), these results show that cell permeation does not provide significant thermal stability in aqueous media relative to the free LipIAF5-2 enzyme [13]. This result contrasts with other reports, where immobilized esterases in an *E. coli* WCB have been shown to display 11-fold increases in enzyme half-life relative to crude extracts of the free enzyme form [28].

### Effect of Water Content and Organic Solvents on the Activity of the LipIAF5-2 WCB

Water content in any organic bio-synthesis is essential for enzyme flexibility and for optimal catalytic activity [17]. The effect of water content on WCB initial activity was examined using water concentrations ranging from 0% to 75% (w/w) in *tert*-butanol. While maximum initial activity was observed at 25% water (not shown), maximum molar conversion was obtained with 10% water content (Fig. 2B). As expected, this observation suggests that hydrolysis competes with organic synthesis at higher water concentrations. Although synthetic activity of most lipases is optimal at relatively low water content in organic systems (typically below 1% (w/w)), previous studies with *E. coli* WCB showed optimal synthesis activity at water contents up to 70% (w/w) [27]. Our results differ from those obtained by Wei and co-workers, owing to the fact that LipIAF5-2 WCB probably needs lower water content to sustain proper conformational flexibility than other intracellular WCB. Similar results were obtained with fungal WCB, albeit for the transesterification of long-chain TAGs [24], [39], [40]. The lower reaction rates observed at higher water contents such as 50 and 75% is probably due to the formation of a bi-phasic system, as the triglycerides are highly insoluble in the excess water phase. Lower water contents also allow for a decrease in the competing hydrolysis reaction. Nevertheless, the fact that the LipIAF5-2 biocatalyst is optimal at a relatively high water content compared to most free and immobilized enzyme systems is interesting, as it would allow the esterification of free fatty acids without the need for *in situ* removal of the water produced during the reaction. Direct esterification of free fatty acids (FFA) in organic media is an important industrial reaction for flavor synthesis. As a result, elaborate strategies must be applied to avoid progressive decreases in rates caused by the production of water, such as water adsorbing materials, pervaporation, gas stripping and continuous distillation [2]. The use of a simple, cheap and water-tolerant WCB for esterification reactions could circumvent the need to implement such cumbersome and expensive methods for the industrial production of flavors.

Synthetic reactions in microaqueous organic media are also influenced by hydrophobicity and polarity of the medium. It is generally accepted that the most suitable co-solvents are the ones with a log *P* (where *P* is the partitioning coefficient of solvent between 1-octanol and water) higher than 2, ideally above 4 [41]. Solvents below these values are known to have a deleterious impact on dehydrated enzymes by stripping the tightly bound water layer from the biocatalyst, resulting in enzyme structure perturbation [17]. Depending on water activity and hydrophobicity of the substrates and products, different organic solvents could even modulate the specificity of the enzyme [17]. To test this, the activity of the WCB was assessed toward transesterification of glyceryl tributyrate with methanol in various water-soluble polar solvents (acetonitrile and *tert*-butanol), hydrophobic polar (ethyl acetate and 2-octanol) and non-polar solvents (*n-*hexane and *n-*heptane). The results are summarized in Table 2. As shown, the presence of polar groups combined to a hydrophobic core seems beneficial to enzyme activity, with the highest activities observed for 2*-*octanol and *tert*-butanol. While water-soluble co-solvents are generally known to be deleterious, the fact that *tert*-butanol is a good medium is interesting and suggests that alcohols employed as substrates could even be suitable solvents for the synthetic reactions. Reports showed that substrate and product hydrophobicity is also very important for the type of solvent suitable to the enzyme [41]. Since *tert*-butanol is miscible with water and all substrates tested, in addition to the fact that it has a lower boiling point–thus easier to strip from the synthesized esters–, it was chosen as the optimal solvent for all further chemical reactions.

**Table 2:** Impact of co-solvents on the initial activity of the LipIAF5-2 WCB.

Solvent	PartitionCoefficient (log *P*)^a^	DielectricConstant (ε)^b^	Initial activity(nmol·min^−1^mg^−1^)	Conversion^c^ (%)
Acetonitrile	−0.34	37.5	0.030	12.8±1.7
Ethyl acetate	0.73	6.0	0.050	16.9±1.9
*tert*-butanol	0.35	10.9	0.090	31.8±2.7
2-octanol	2.9	10.3	0.170	67.9±0.5
*n*-hexane	4.0	1.9	0.050	21.8±2.7
*n*-heptane	4.5	1.9	0.060	24.9±1.2

a Taken from [35].

b Taken from [30].

c Conversion is expressed as a percentage of maximal theoretical yield after 60 minutes of reaction under standard assay conditions.

### Impact of LipIAF5-2 WCB Loading on Ester Synthesis

The influence of biocatalyst loading on the production of methyl butyrate was also examined. As show in Figure 2C, a direct correlation between molar conversion of glyceryl tributyrate and increased WCB concentration was observed. The conversion of 1 mmol of glyceryl tributyrate after a 3-hour incubation increased from 12.5% to 70.6% when the amount of biocatalyst increased from 5 mg to 50 mg. Further increasing cell loading in a fixed-volume vessel was not possible without impairing sampling, since the reaction mixture volume occupied by the biocatalyst was greater than the total volume available. Increasing the total reaction volume to a bigger vessel did, however, reduce WCB synthesis rates, probably due to dilution of the reactants with additional co-solvent. Nevertheless, our maximal ester synthesis yield was observed at only 6% WCB loading (w/w). This is much lower than the previous 30% (w/w) ratio reported for optimal transesterification of olive oil [27], yet very similar to the optimal 4% (w/w) ratio observed in a recent study on grease transesterification [42]. A solution to the issue of the volume occupied by the WCB would be to use a fluidized bed reactor (FBR), where cells act as a matrix trapped inside a column and the reaction mixture is pumped through the cells (*vide infra*). This type of bioreactor minimizes the clogging and backpressure issues often observed with packed bed reactors, in addition to easing the recycling of the catalyst as compared with most batch reactors [43], [44].

### Effect of Alcohol Concentration on LipIAF5-2 WCB

Biosynthesis of alcohol esters is often limited by substrate concentrations, where high amounts of water-soluble alcohols denature the biocatalyst by interfering with the enzyme-bound water layer [45]. For the transesterification of TAGs, it is often necessary to add the alcohol in a stepwise manner to keep the actual concentration below the stoichiometric ratio of 3∶1 (alcohol:substrate) [46]. The protective environment produced by the cell could therefore protect the lipase from this stripping effect. Contrary to most lipases, LipIAF5-2 showed increased activity with increasing molar ratios of acyl acceptor, with a maximal velocity observed at a molar ratio of 10∶1 (methanol:glyceryl tributyrate) (Fig. 2D). This represents a concentration of 27% (v/v) methanol in the reaction vessel, illustrating that the LipIAF5-2 WCB has a very good resistance to alcohol inhibition. Wei and co-workers did report the highest WCB activity at ratios of 5∶1, but only with water content between 50 and 100% (w/w) [27]. To the best of our knowledge, this is the first report of such high methanol tolerance for a lipase biocatalyst, a property that could prove very useful to avoid stepwise addition of methanol during biosynthesis of various methyl esters.

### Impact of Acyl Donor on Ester Synthesis

**Figure 3:**
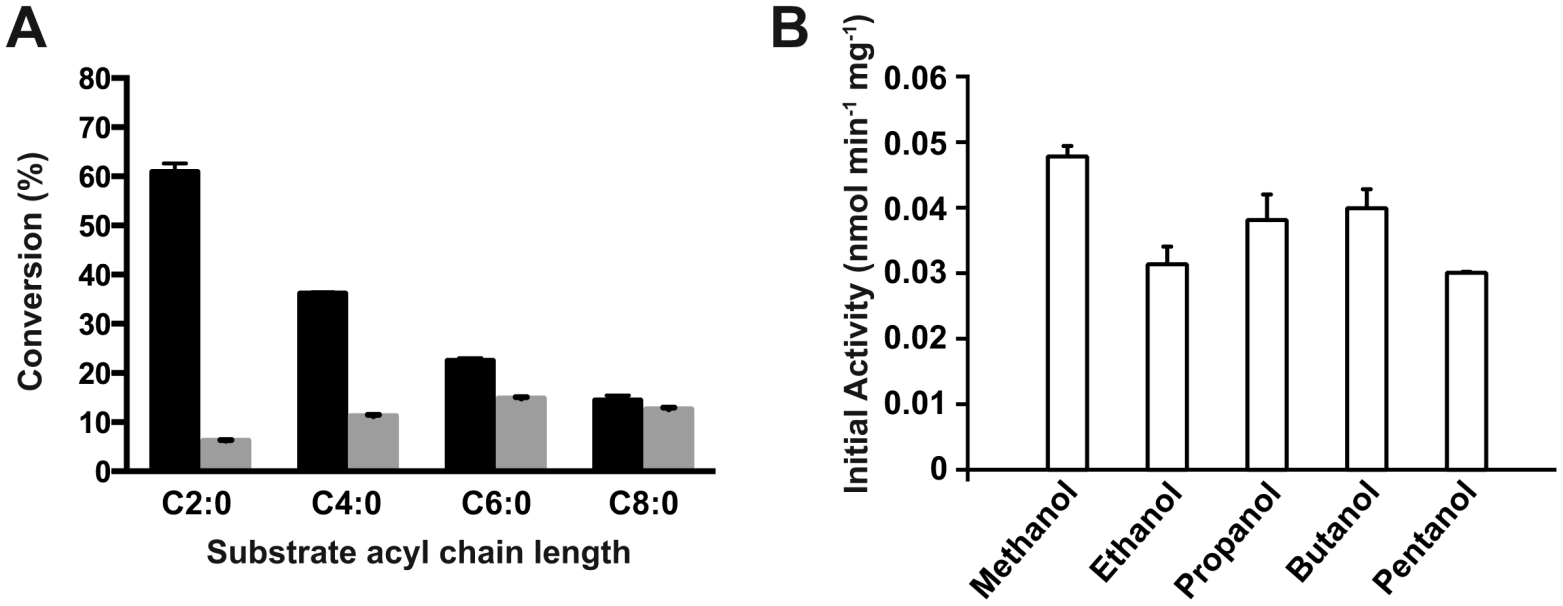
Conversion rates of LipIAF5-2 WCB transesterification and esterification toward acyl donors and acceptors. A) The full bars represent the conversion of triglyceride substrates and the shaded gray bars the conversion of equivalent free fatty acids (FFA). The substrates employed are glyceryl triacetate and acetic acid (C2∶0), glyceryl tributyrate and tributyric acid (C4∶0), glyceryl trihexanoate and hexanoic acid (C6∶0) and glyceryl trioctanoate and octanoic acid (C8∶0). The results are expressed as the molar conversion of 1 mmol of each substrate after 6 hours of incubation at 40°C with 4 molar equivalents of methanol and 10 mg of LipIAF5-2 WCB. B) Impact of acyl acceptors on transesterification by LipIAF5-2 WCB. Transesterification of 1 mmol of glyceryl tributyrate was done using 4 molar equivalents of each primary alcohols for 3 hours at 40°C using 10 mg of LipIAF5-2 WCB and 10% (w/w) water. Results are averaged over two independent experiments performed in triplicate.

The specificity of the WCB toward various TAGs and equivalent FFA as acyl donors was assessed for the transesterification and esterification reactions, respectively (Fig. 3A). Results showed a remarkable difference between the specificity of the enzyme for the transesterification and esterification reactions. While transesterification is favored by short acyl donor chains, esterification is compromised by acetic acid (C2∶0). Numerous studies reported potent enzyme inhibition by very short FFA acyl donors [14], [47], [48]. One explanation is the possible dead-end inhibition conferred by the carboxylic acid reacting with the active site serine residue of the hydrolase [48]. Furthermore, esterification is much slower than transesterification in the conditions tested, suggesting that LipIAF5-2 has higher affinity for glycerol esters. Esterification with 0.5 M FFA instead of 1 M produced the same conversion yields (data not shown). However, this shows that the LipIAF5-2 WCB is tolerant to high concentrations of FFA relative to other immobilized lipases under similar conditions [32], [49]. To assess the impact of the acyl acceptor, the synthetic activity of the WCB was performed using various primary alcohols (Fig. 3B). Results show that methanol is the best acyl acceptor for LipIAF5-2 WCB. Nevertheless, the enzyme could efficiently substitute methanol for ethanol, propanol, butanol or pentanol, demonstrating the high potential of the LipIAF5-2 WCB for the synthesis of most short-chain flavor esters.

### Repeated-batch Transesterification by LipIAF5-2 WCB

**Figure 4:**
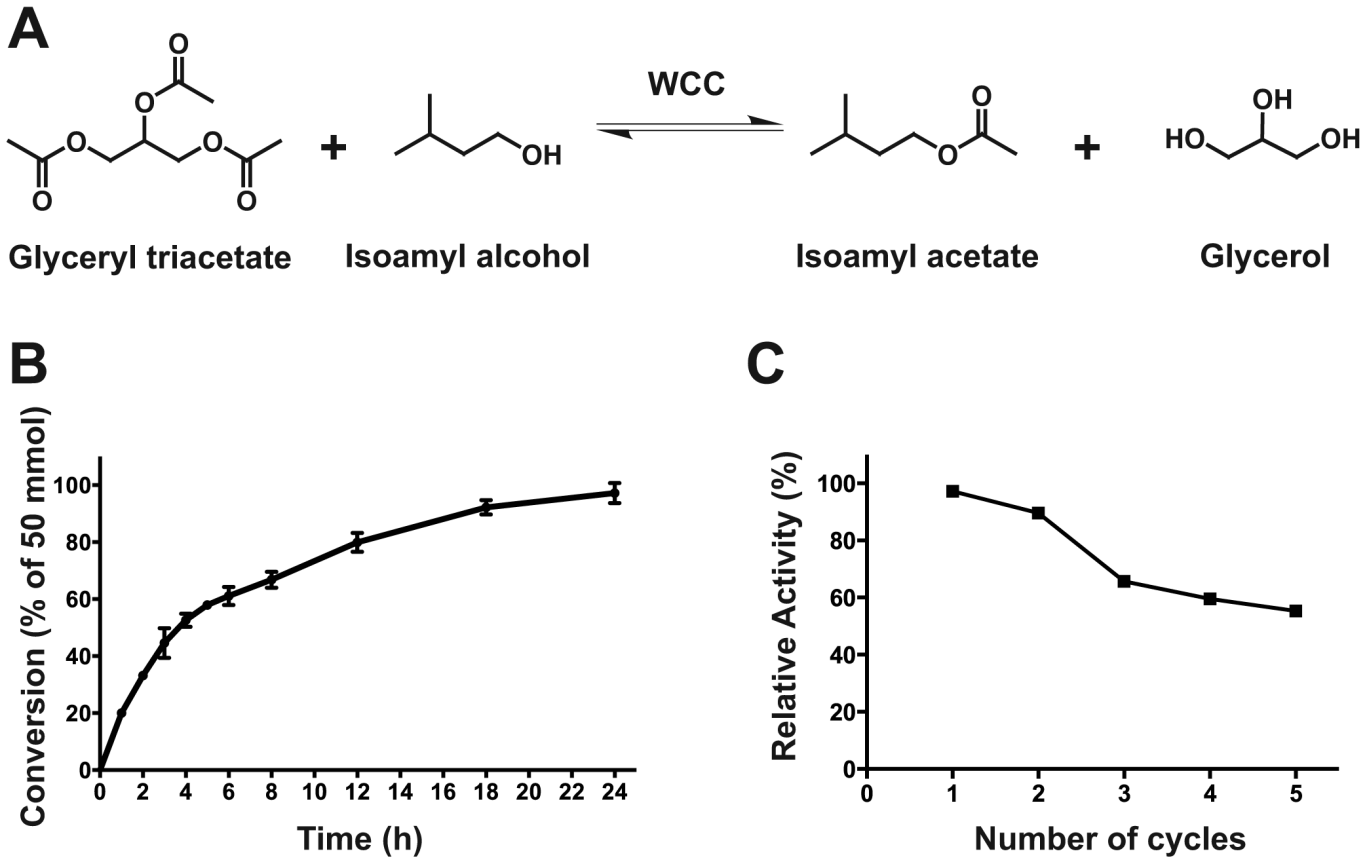
Time-course conversion of glyceryl triacetate into isoamyl acetate using the LipIAF5-2 WCB. A) Reaction scheme for the transesterification of glyceryl triacetate into isoamyl acetate in presence of isoamyl alcohol. B) Time-course production of isoamyl acetate by LipIAF5-2 WCB immobilized in a fluidized bed reactor. C) Effect of LipIAF5-2 WCB recycling on the conversion yield of isoamyl acetate production. One gram of LipIAF5-2 WCB was loaded in an empty HPLC preparative column with a dead volume of 10 cm^3^ and placed in a heated chamber. The reaction mixture contained 50 mmoles of glyceryl triacetate as acyl donor and isoamyl alcohol at a molar ratio of 5∶1 with 5% water (w/w). The mixture was pumped in recirculating mode through the column at a flow rate of 1 mL/min for 24 h for each cycle. Cells were washed in open mode with 350 mL of *tert*-butanol between each cycle. All experiments were performed in triplicate.

To demonstrate the feasibility of flavor ester production using the optimized synthetic conditions, a FBR was built using an empty preparative HPLC column filled with the LipIAF5-2 WCB (Fig. S2). The model reaction selected was the transesterification of glyceryl triacetate into the important banana fragrance and pear-drop ingredient isoamyl acetate (Fig. 4A). Using 1 g of WCB for 50 mmoles of substrate (biocatalyst loading ratio of 9.2% w/w), time-course reactions showed a maximal conversion yield of 97.2±3.3% after 24 hours of incubation in recirculating mode (Fig. 4B). Using the adequate continuous flow in an open circuit (*i.e.* non-recirculating), one could expect to target the saturation point of the biocatalyst, where a production of 3.9 g of isoamyl acetate per hour was achieved with 1 gram of WCB. This demonstrates a very interesting production rate, as the synthesized flavor can easily be purified using simple distillation procedures. Furthermore, immobilization of the WCB on a HPLC column allowed recycling of the biocatalyst after an easy washing step with *tert*-butanol. Using these conditions, the LipIAF5-2 WCB could be efficiently recycled up to 5 times without any cells loss, albeit with a significant decrease in production yields (55.3±0.3% after 5 cycles at 35°C) (Fig. 4C). This efficiency drop could either be caused by enzyme denaturation after prolonged exposure at 35°C and/or a direct consequence of enzyme leaching from the cells. Previous reports on WCB did highlight this phenomenon in similar *tert*-butanol organic systems [50]. Among the suggested improvements to help minimize enzyme leaking, covalent binding of the enzyme within the cell using glutaraldehyde was suggested [51]. Alternatively, enzyme immobilization at the cell surface using natural membrane anchors could represent a powerful alternative, thus avoiding the permeation step required to reach significant synthesis rates [32], [52], [53]. Such immobilization is currently under investigation to improve the industrial potential of the LipIAF5-2 WCB.

## Conclusions

In the present work, we have shown that an *E. coli* recombinant system expressing the novel lipase LipIAF5-2 extracted from a metagenomic study could easily be used as an efficient whole-cell biocatalyst for the synthesis of short-chain flavor esters. Following a simple permeation step, the WCB showed high conversion yields and good recycling properties in micro-aqueous media. Furthermore, the biocatalyst displayed high versatility toward short-chain precursors typical of flavor esters, combined to remarkable resistance to fatty acid and alcohol denaturation. These results demonstrate a promising potential for the industrial biosynthesis of relevant short-chain flavor esters under mild conditions.

## Supporting Information

Figure S1 **SDS-PAGE (A) and zymogram (B) of purified recombinant LipIAF5-2.** Lane 1: Precision Plus All Blue molecular weight standard (Bio-Rad). Lane 2: Soluble fraction of control strain harboring vector pET16b. Lanes 3: soluble fraction of *E. coli* strain harboring pET16b-F52. Lane 4: Purified LipIAF5-2 after IMAC chromatography. The equivalent of 5 μg of purified enzymes were loaded in lane 4.
(TIF)

Figure S2 **Fluidized bed reactor scheme.** See Materials and Methods for details.
(TIF)
