# Supplementary figures and images for: Short-Chain Flavor Ester Synthesis in Organic Media by an E. coli Whole-Cell Biocatalyst Expressing a Newly Characterized Heterologous Lipase
Source: PLoS One. 2014 Mar 26;9(3):e91872. doi: 10.1371/journal.pone.0091872 (PMC3966760; doi:10.1371/journal.pone.0091872)

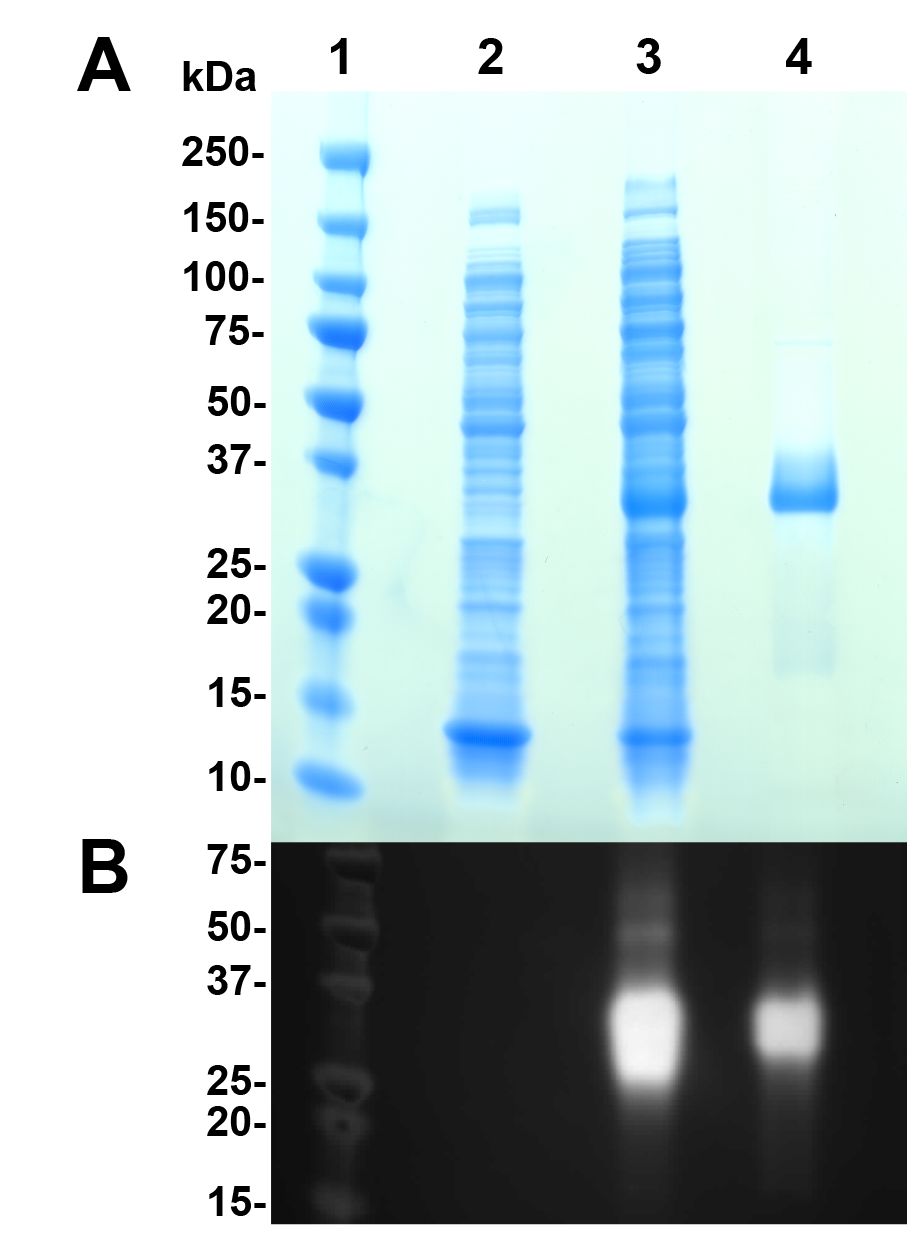

Supplement: Figure S1 — SDS-PAGE (A) and zymogram (B) of purified recombinant LipIAF5-2. Lane 1: Precision Plus All Blue molecular weight standard (Bio-Rad). Lane 2: Soluble fraction of control strain harboring vector pET16b. Lanes 3: soluble fraction of E. coli strain harboring pET16b-F52. Lane 4: Purified LipIAF5-2 after IMAC chromatography. The equivalent of 5 μg of purified enzymes were loaded in lane 4. (TIF) [file pone.0091872.s001.tif]

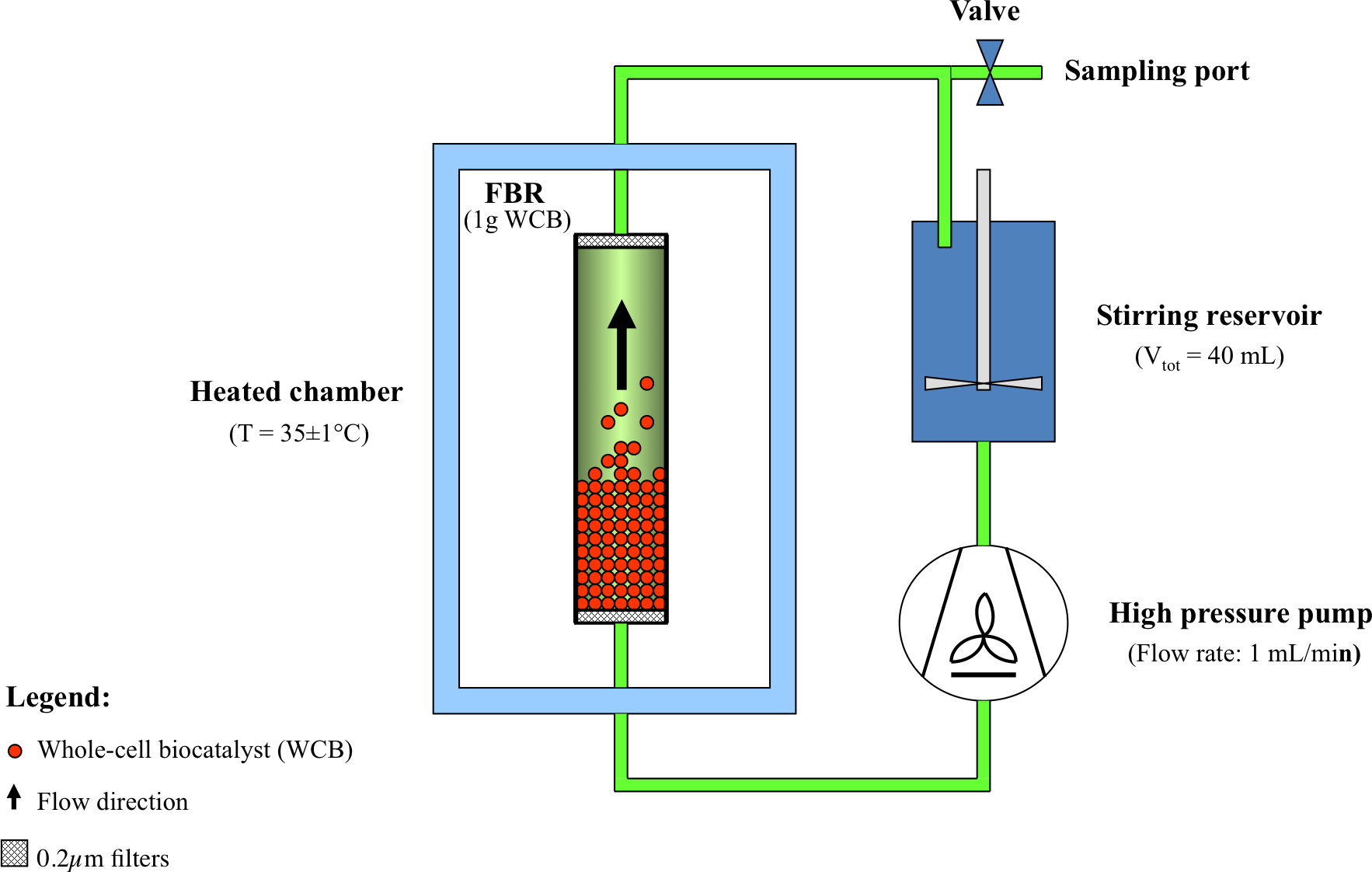

Supplement: Figure S2 — Fluidized bed reactor scheme. See Materials and Methods for details. (TIF) [file pone.0091872.s002.tif]
